# A Reassessment of the Impact of European Contact on the Structure of Native American Genetic Diversity

**DOI:** 10.1371/journal.pone.0161018

**Published:** 2016-08-31

**Authors:** Keith Hunley, Kiela Gwin, Brendan Liberman

**Affiliations:** Department of Anthropology, University of New Mexico, Albuquerque, NM, 87131, United States of America; Universitat Pompeu Fabra, SPAIN

## Abstract

Our current understanding of pre-Columbian history in the Americas rests in part on several trends identified in recent genetic studies. The goal of this study is to reexamine these trends in light of the impact of post-Columbian admixture and the methods used to study admixture. The previously-published data consist of 645 autosomal microsatellite genotypes from 1046 individuals in 63 populations. We used *STRUCTURE* to estimate ancestry proportions and tested the sensitivity of these estimates to the choice of the number of clusters, *K*. We used partial correlation analyses to examine the relationship between gene diversity and geographic distance from Beringia, controlling for non-Native American ancestry (from Africa, Europe and East Asia), and taking into account alternative paths of migration. Principal component analysis and multidimensional scaling were used to investigate the relationships between Andean and non-Andean populations and to explore gene-language correspondence. We found that 1) European and East Asian ancestry estimates decline as *K* increases, especially in Native Canadian populations, 2) a north-south decline in gene diversity is driven by low diversity in Amazonian and Paraguayan populations, not serial founder effects from Beringia, 3) controlling for non-Native American ancestry, populations in the Andes and Mesoamerica have higher gene diversity than populations in other regions, and 4) patterns of genetic and linguistic diversity are poorly correlated. We conclude that patterns of diversity previously attributed to pre-Columbian processes may in part reflect post-Columbian admixture and the choice of *K* in *STRUCTURE* analyses. Accounting for admixture, the pattern of diversity is inconsistent with a north-south founder effect process, though the genetic similarities between Mesoamerican and Andean populations are consistent with rapid dispersal along the western coast of the Americas. Further, even setting aside the disruptive effects of European contact, gene-language congruence is unlikely to have ever existed at the geographic scale analyzed here.

## Introduction

Our current understanding of pre-Columbian history in the Americas rests in part on three trends identified in recent genetic studies. The first trend is a negative correlation between population-level genetic diversity and geographic distance from the Bering Strait, which Wang et al. [1] attributed to a north-south serial founder effect process. This finding was subsequently replicated in a study of Native American mtDNA and Y-chromosome variation [2], and it is consistent with results from a large-scale study of autosomal SNP diversity in native Mexican populations [3]. Furthermore, when coastlines were treated as preferred routes as compared to direct great-circle distances, Wang and colleagues found that the magnitude of the correlation between heterozygosity and distance from Beringia increased (from r = -0.436 to -0.585), suggesting that the initial movement into the Americas occurred mainly along the coasts.

In a subsequent study of the same data, Hunley and Healy (2011) found that the level of European ancestry in the 29 Native American populations was also negatively correlated with geographic distance from Beringia. Moreover, after controlling for European ancestry in partial correlation analyses, they demonstrated that the magnitude of the correlation fell dramatically and lost statistical significance. This finding potentially undermines the role of serial founder effects in shaping patterns of Native American diversity. However, Hunley and Healy failed to consider the possibility that more than three ancestral groups contributed to extant admixed Native American populations (African, European, and Native American). In particular, they failed to consider potential contributions from East Asian populations that may have occurred subsequent to initial peopling [1,4–6]. To the extent that allele frequencies are correlated between European and East Asian populations, this exclusion may have resulted in overestimation of European ancestry in Native American populations.

The second trend is that Western South American populations have higher diversity than populations in eastern portions of the continent [1,7–9]. In combination with the finding that Andean populations are relatively undifferentiated from Mesoamerican populations [1], these results potentially support the hypothesis of coastal colonization of the Andes from Mesoamerica, followed by dispersal from the Andes into Eastern South America. However, a study of mtDNA d-loop sequence variation by Lewis and Long [10] demonstrated that diversity varied substantially within and between Western and Eastern South American populations [11]. Furthermore, Andean populations have higher European ancestry than Amazonian populations [12], raising the possibility that high Andean diversity is the result of post-Columbian admixture.

The third trend is a correspondence between patterns of genetic and linguistic variation [1,13–17]. Many studies that identify gene-language correspondence rely on Greenberg’s language classification [18], which is broadly rejected by Native American language specialists [19–25]. One of the many points of contention is that Greenberg created language groups based in part on similarities that were due to borrowing between genealogically unrelated languages [25]. This approach potentially conflates geographic and linguistic proximity and may lead to overestimation of the degree of gene-language correspondence. Additionally, because admixture affects gene diversity within and between populations, it has the potential to also affect the relationship between genetic and linguistic distances. For these reasons, the true degree of gene-language correspondence in the Americas, and its potential evolutionary causes, remains uncertain.

The goal of this study is to reexamine these trends in the pattern of Native American diversity in light of the limitations imposed by the admixture process itself and by the data and methods used to measure the contribution of ancestral sources to admixed populations. We concentrate on four questions that are commonly addressed in genetic studies of Native American prehistory:

1. What is the level of African, European, and East Asian ancestry in Native American populations?
2. What routes did people take during their initial dispersal across the Americas?
3. How are native Andean populations related to populations from other regions of the Americas?
4. Are patterns of population genetic and linguistic variation correlated?

## Materials and Methods

### Data

The genetic data come from published sources. They consist of 645 autosomal microsatellite genotypes from 1046 individuals in 63 populations (7 African, 8 European, 19 East Asian, and 29 Native American). Microsatellite data are well-suited for this study because they are relatively free of ascertainment bias and therefore provide accurate estimates of gene diversity. The data were compiled by Pemberton et al. [26] from Rosenberg et al. [27,28] and Wang et al. [1]. In compiling the data, Pemberton et al. excluded one individual from each pair of monozygotic twins and first degree relative pairs, excluding the Karitiana and Suruí [28].

For most analyses, we labeled the Native American populations as belonging to one of nine geographic regions, initially defined based on geographic location and subsequently refined based on results of our analyses. The regions are: Canada (n = 3), northern Mexico (n = 1, Pima), Mesoamerica excluding Costa Rica (n = 5), Costa Rica and Panama (n = 2), coastal Colombia (n = 6), inland Colombia (n = 2), the Andes (n = 3), Amazonia and Paraguay (n = 5), and Southern Brazil (n = 2).

### Genetic ancestry

We used the model-based clustering approach implemented in *STRUCTURE* [29] to estimate African, European, East Asian and Native American ancestry in individuals and populations. We used the approach described by Evanno et al. [30] to assist in identifying *K*. The approach uses a statistic (Δ*K*) related to the second order rate of change in the posterior probability of the data given *K* (see Discussion). Based on Evanno’s et al. analysis of a simulated hierarchical island model, we first ran *STRUCTURE* on the full 63-population dataset for values of *K* from 5–21, and we then ran *STRUCTURE* for the 29 Native American populations only at values of *K* from 5–28. For each *K*, we ran *STRUCTURE* a minimum of 15 times using a burnin phase of 25,000 steps and 15,000 MCMC repetitions. Otherwise, we used the default settings in *STRUCTURE*, including the admixture model and the correlated-allele-frequency model, and we allowed the degree of admixture among populations to be inferred from the data. We displayed the *STRUCTURE* results using bar charts and violin plots (combined boxplots and rotated kernel density plots) [31].We used the Structure Harvester program [30] to calculate Δ*K* and related statistics.

#### Sensitivity of ancestry estimates to the choice of K

To assess the effects of *K* on ancestry estimates, we averaged the African, European, East Asian, and Native American ancestry estimates in each population for each value of *K* (across 15+ runs), and displayed the results using line graphs.

### Gene diversity vs. geographic distance from Beringia

We constructed scatter plots of gene diversity [32] vs. geographic distance from Beringia. We initially computed great circle distances from Beringia to each Native American population using coordinates provided by Wang et al. [1]. We then computed distances along alternative paths that populations might have followed during their initial dispersal. We considered movement from Beringia along coasts, e.g., along the north coast of Canada for the Native Canadian populations, along the Pacific coast for Mesoamerican and Andean populations, and along the Atlantic coast of South American for the Amazonian, Paraguayan and southern Brazilian populations. For the Amazonian populations, we also considered paths along the Amazon River. For the Uto-Aztecan-speaking Pima population, we considered a path from Beringia to Mesoamerica and then back northward to their current location, in keeping with the farming-language dispersal hypothesis for the spread of Uto-Aztecan languages [33]. While the various paths affected the magnitude of the correlation between gene diversity and distance from Beringia, the correlation was statistically significant for all paths, and our interpretation of the correlations is unaffected by the best-fit path reported below.

To assess and control for the effects of non-Native American admixture (African, European, and East Asian), we performed partial correlation analyses between gene diversity and geographic distance controlling for combined African, European, and East Asian ancestry, referred to here as “non-Native American ancestry.” We report the partial correlation coefficients and display the results of the partial correlation analyses using a scatter plot of the residuals of linear models of non-Native American ancestry and gene diversity vs. non-Native American ancestry and geographic distance. We also used a jackknife approach to identify the contribution of each population and region to the magnitude of the correlation and partial correlation between gene diversity and distance from Beringia. For these analyses, we dropped each population (and region) from the analysis, one at a time, and then re-computed the correlation.

### Principal Component Analysis

We used principal component analysis (PCA) to summarize the major axes of genetic variation in the full 63-population dataset. We extracted the first 25 PCA factors, first scaling the allele frequencies using the scaleGen function from the adegenet package in R [34], and then performing the PCA analysis using the dudi.pca function from the ade4 package [35]. We displayed the first six factors using boxplots. Because the first three PCA factors were correlated with European ancestry in the Native American populations and the first six PCA factors were influenced by genetic substructure in Africa, we repeated the PCA analyses using just the 29 Native American populations.

### Language

We used the Ethnologue [36], in conjunction with Campbell [25] to identify the language families for the Native American populations. Importantly, for our analyses, we did not consider unattested higher-level groupings of these families [25], meaning that we did not consider relationships between the language families.

We replotted the PCA factors from the Native American-only analyses described above, color-coding the populations by language family affiliation. We also constructed multidimensional scaling (MDS) plots from Nei’s minimum genetic distances [37] for the full 63-population sample and separately for the 29 Native American populations. We repeated the analyses using other measures of genetic distance, most notably (δμ)^2^ [37], which takes into account the stepwise mutation model of microsatellite evolution. The different measures were strongly correlated with one another, e.g., r_Nei-(δμ)2_ = 0.945 for the Native American populations. The results and interpretations that we report below were unaffected by the choice of the distance measure. In the plot of the first two principal coordinates (PCos) for the Native American-only sample, we color-coded the points by language family and scaled the point size according to the level of non-Native American ancestry.

Analyses and plotting were carried out using the base R package and the packages ade4, ppcor, fields, vegan, vioplot, and RcolorBrewer [31,38–41].

## Results

### Genetic ancestry

**Fig 1:**
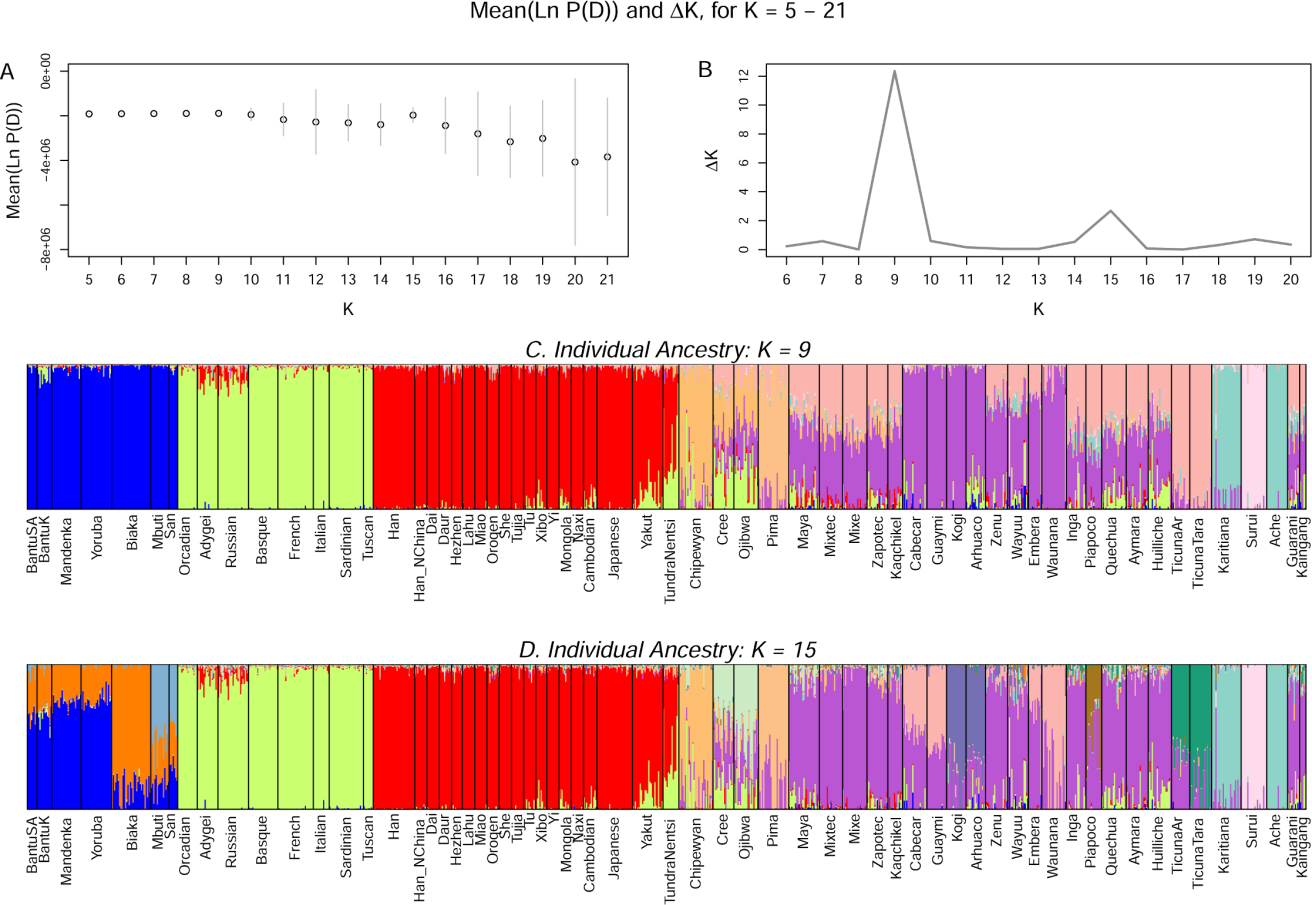
**A. Mean log probability of data +/- 1 SD for *K* = 5–15. B.** Δ***K*. C. Bar chart of individual ancestry for a typical run at *K* = 9. D. Bar chart of individual ancestry for a typical run at *K* = 15.**

The results of the *STRUCTURE* analyses are consistent with those from previous studies in showing the existence of geographically-patterned genetic structure at all values of *K* [1,27]. The mean log probability of data for 15 runs at each value of *K* increased steadily from *K* = 5 to *K* = 9, after which it decayed, with the exception of a spike at *K* = 15 (Fig 1A). The ΔK statistic also showed a sharp peak at *K* = 9 and a lower peak at *K* = 15 (Fig 1B).

Fig 1C and 1D shows bar charts of ancestry estimates for typical runs at *K* = 9 and *K* = 15. The two runs are distinguished by increasing substructure within Africa and the Americas. In Africa, three region-specific clusters exist at *K* = 15 compared to a single cluster at *K* = 9. In the Americas, at *K* = 9, region-specific clusters exist for the three Canadian populations, the Pima, the Suruí, and the Karitiana-Ache. At *K* = 15, the Canadian-specific ancestry component separated into Chipewyan- and Cree-Ojibwa-specific components, and new region-specific ancestry components formed in the Kogi-Arhuaco, Piapoco, Ticuna Arara-Ticuna Tarapaca, and the Cabécar-Guaymi-Emberá-Waunana. Additional region-specific structure formed at higher values of *K*.

At *K* = 9, the mean African and East Asian ancestry estimates in the Native American populations were relatively low at 0.9% and 1.5% respectively. In contrast, the mean European ancestry was relatively high at 6.8%, and it exceeded 10% in 8 populations. At the regional level, it was highest in Canada (mean = 16.7%) and the Andes (mean = 9.9%) and lowest in Amazonia (mean = 0.02%). The mean for the largest Native American-specific ancestry component, which was shared by all Native American populations, shown in purple, was 67.5%. We hereafter refer to this general Native American-specific ancestry component as “pan-Native American” ancestry

At *K* = 15, the mean African ancestry across the populations was essentially unchanged at 1.0%, but the mean East Asian ancestry fell to 0.8%, the mean European ancestry fell to 4.9%, and the mean pan-Native American ancestry fell to 50.3%. These estimates continued to fall at even higher values of *K*. These results indicate that ancestry estimates in Native American populations are potentially sensitive to the choice of *K*.

### The effect of K on ancestry estimates

**Fig 2:**
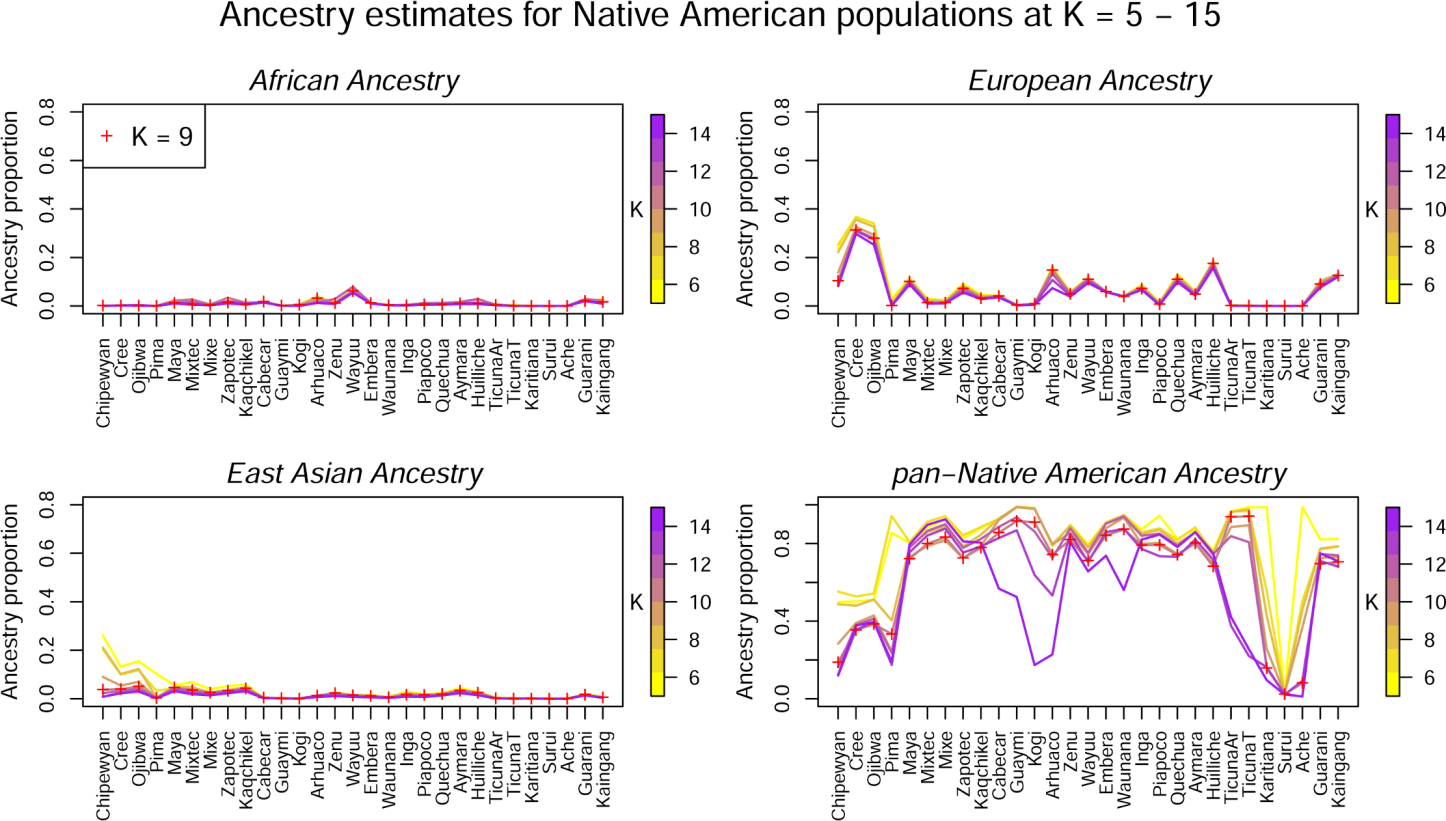
Ancestry estimates within each population at values of *K* from 5–15. The lines are color coded according to the values of *K*, with low values in yellow and high values in purple. The red ‘+’ signs show the estimates at K = 9.

To further explore the sensitivity of ancestry estimates to the choice of *K*, we plotted the mean ancestry for each population for each value of *K* in Fig 2. The plot shows that African ancestry is relatively uniform in Native American populations for all values of *K*. In contrast, European ancestry decreased in all populations as *K* increased. The decrease leveled off in some cases, but in others cases, it continued through *K* = 21 (the highest value of *K* that we analyzed). The most notable decrease occurred in the three Canadian populations and the Colombian Arhuaco population. East Asian ancestry also decreased steadily in the Canadian populations from *K* = 5 to *K* = 9, at which point it stabilized at a mean of about 1%. The pan-Native American ancestry component also decreased as *K* increased; these changes reflect the continual formation of Native American region-specific ancestry clusters.

**Fig 3:**
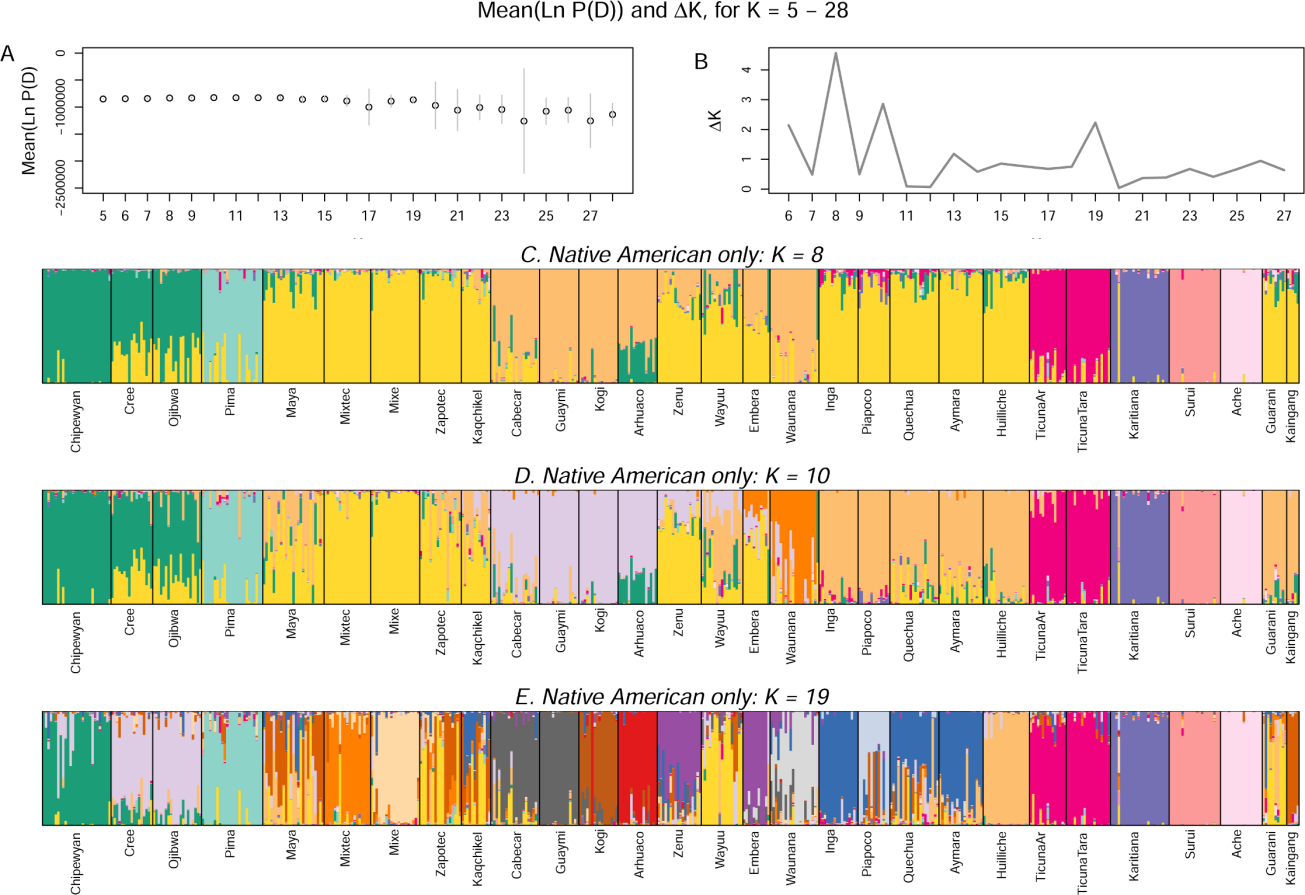
**A. Mean log probability of data +/- 1 SD for analyses restricted to the Native American populations at *K* = 5–28. B.** Δ***K*. C—E. Bar charts of individual ancestry for typical runs at *K* = 8, 10, and 19. **

To further explore genetic substructure in the Americas, we also performed *STRUCTURE* analyses of the 29 Native American populations alone (Fig 3). These analyses are broadly consistent with those from the analysis of the full 63-population dataset. This time, however, the mean log probability of data and Δ*K* were less informative about the value of *K*, largely because of increased variance across runs above *K* = 15. Δ*K* peaked at *K* = 8, but it also showed strong peaks at *K* = 10 and 19 and weaker peaks at other values of *K*.

As was the case with the full sample, region-specific clusters formed as *K* increased. At *K* > 19, several regional clusters further divided into population-specific clusters. As with the 63-population analyses, the Native American region-specific ancestry components that formed at higher values of *K* did so at the expense of a pan-Native American ancestry component.

### What routes did populations take during their initial dispersal across the Americas?

**Fig 4:**
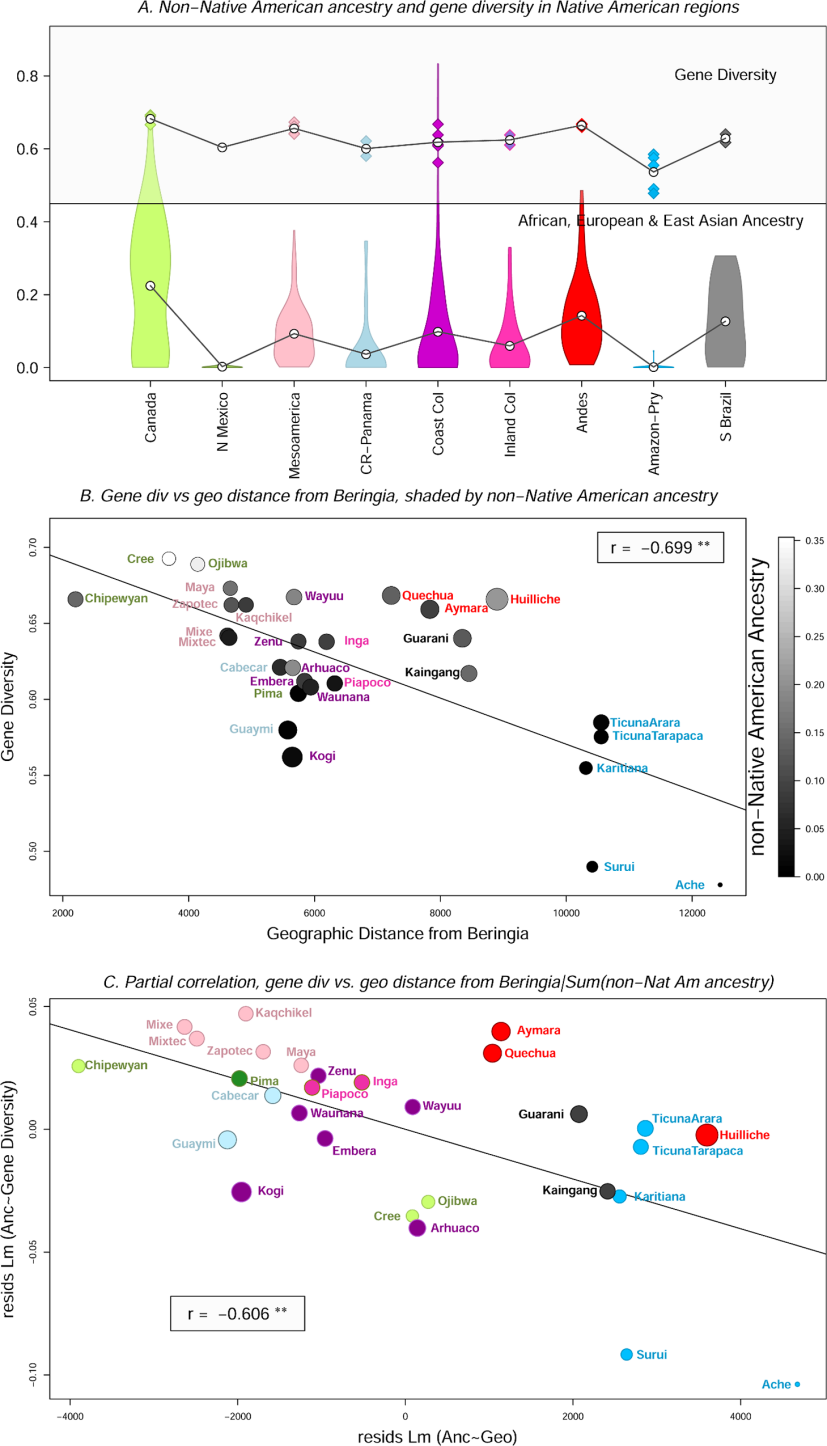
A. Violin plots of non-Native American ancestry (at *K* = 9, bottom) and gene diversity (top), in each of the nine Native American regions. B. Gene diversity vs. geographic distance from Beringia for best-fit paths of movement. Population names are colored according to geographic region, using the same colors shown in A. The size of each point shows the effect of removing that population from the analysis (jackknife analyses). Large size indicates that the removal of the population increased the magnitude of the correlation (more negative *r*), and small size indicates that removal of the population reduced the magnitude of the correlation. The points are color-coded by non-Native American ancestry, with lighter shades of gray representing higher values. ** indicates *p* < 0.005 C. Graphical representation of the partial correlation analyses controlling for non-Native American ancestry. The y-axis is the residual scores for the linear model of non-Native American ancestry vs. gene diversity. The x-axis is the residual scores for the linear model of non-Native American ancestry vs. geographic distance. Population names and points are color-coded by geographic region, and the size of the points reflects the results of the jackknife results for the partial correlation analyses.

The violin plots in Fig 4A show the distributions of combined African, European, and East Asian ancestry in each Native American region for *K* = 9. The points at the top of the plot show the gene diversity for the populations in each region and the average gene diversity for each region. The plot captures the strong correlation between non-Native American ancestry and gene diversity (*r* = 0.73, *p* < 0.001).

Fig 4B is a scatter plot of gene diversity vs. adjusted geographic distance from Beringia. While all paths that we considered produced statistically significant correlations, the adjusted distances that produced the strongest correlation included north-coast paths for the Native Canadian populations, a path through Mesoamerica for the Pima, Amazonian paths for the Brazilian isolates, and a South American Atlantic coastal path for the Ache.

There are three salient features of the pattern of diversity. First, the correlation between gene diversity and geographic distance from Beringia is high and statistically significant (*r* = -0.699, *p* < 0.000). Second, when populations with high or low non-Native American ancestry are removed from the analysis, the magnitude of the correlation changes. In cases where admixed populations are located far from Beringia (e.g., the Andean populations), their removal strengthens the correlation (makes *r* more negative). Conversely, in cases where admixed populations are located close to Beringia (e.g., the Cree and Ojibwa), their removal reduces the magnitude of the correlation (makes *r* closer to zero). Third, the Amazonian and Paraguayan populations have particularly low diversity, and they have essentially zero non-Native American ancestry. The regional jackknife results in Table 1 show that the overall correlation between gene diversity and geographic distance from Beringia is driven by this low diversity. When the Amazonian and Paraguayan populations are removed from the analysis, the correlation drops and becomes non-significant. These results indicate that the correlation between gene diversity and distance from Beringia may be driven in part by geographically-patterned admixture.

**Table 1:** Region-level Jackknife Results.

	Correlation		Partial Correlation	
Region Dropped	r	p-value	r	p-value
Canada	-0.649	*	-0.609	*
Northern Mexico (Pima)	-0.706	*	-0.701	*
Mesoamerica	-0.668	*	-0.660	*
Costa Rica-Panama	-0.726	*	-0.724	*
Coastal Colombia	-0.748	*	-0.772	*
Inland Colombia	-0.700	*	-0.697	*
Andes	-0.802	*	-0.807	*
Amazonia & Ache	-0.217	0.307	-0.248	0.240
Southern Brazil	-0.722	*	-0.711	*
Average	-0.660		-0.659	

*p < 0.001

To assess the impact of admixture on the correlation, we performed partial correlation analysis of gene diversity vs. distance from Beringia controlling for non-Native American ancestry. Fig 4C graphically shows the results of the partial correlation analysis. The magnitude of the partial correlation (*r*.partial = -0.606, *p* < 0.000) decreased only slightly from the initial correlation, but the pattern of lack of fit is different in that the highest level of diversity is now in Mesoamerica and the Andes, not in Canada. This change is due to the drop in ancestry-corrected diversity in the Canadian Ojibwa and Cree. Another important feature of the plot is that Amazonian and Paraguayan populations are still driving the correlation. When they are removed from the analysis, the partial correlation drops substantially and becomes non-significant (Table 1). We note that we found the same pattern when we estimated non-Native American ancestry using other values of *K*, and when we used other paths of movement to calculate geographic distance from Beringia.

**Fig 5:**
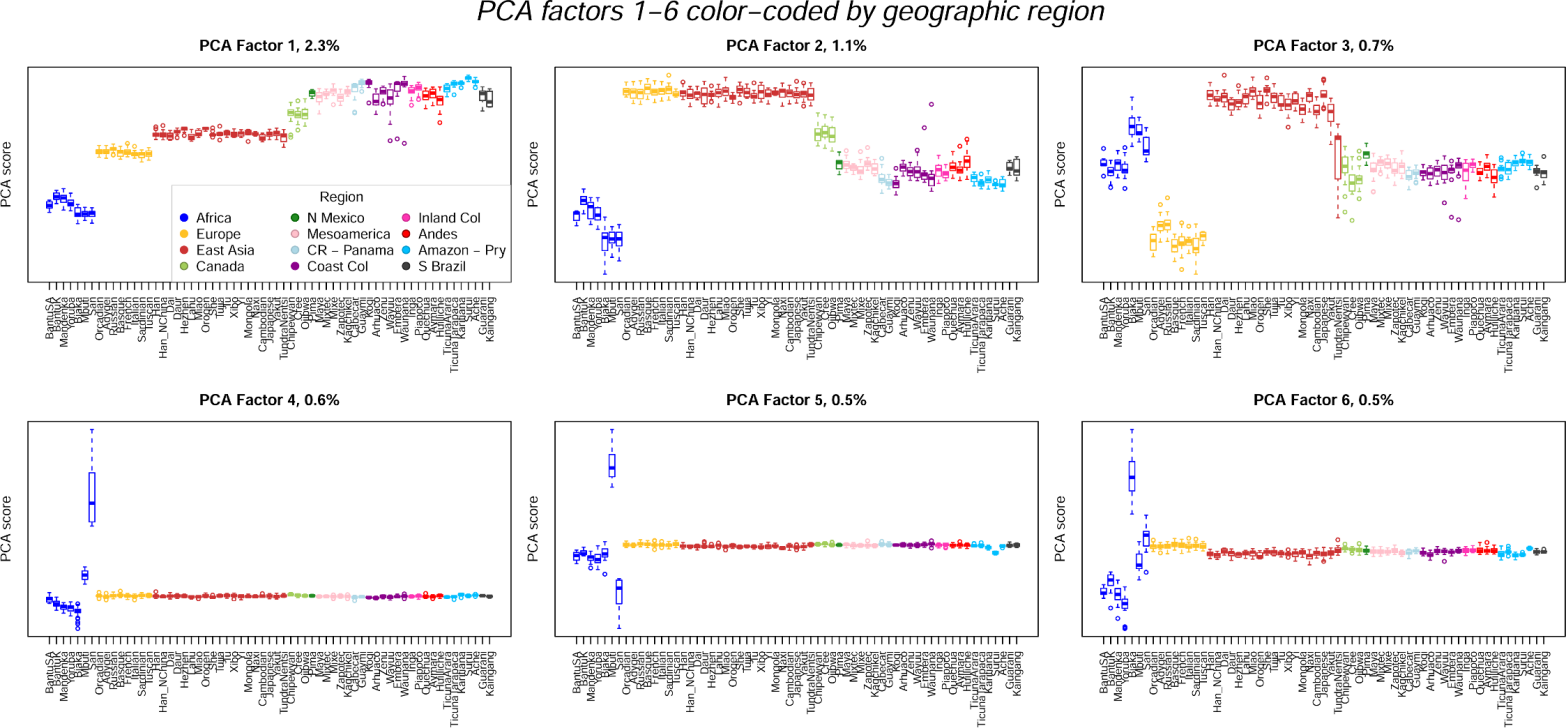
Boxplots of PCAs 1–6 color-coded by geographic region.

To explore these patterns of diversity in more detail, we used principal components analysis (PCA) to summarize the major axes of genetic variation in the full 63-population sample. PCs 1–3, shown using box plots in Fig 5, account for 4.1% of the variation. They separate the major geographic regions in a manner that is consistent with an out-of-Africa serial founder effect process. They also separate one or more of the three native Canadian populations from the other Native American populations, and all three PCs are correlated to varying degrees with European ancestry in the Native American populations (*r*_PC1_ = 0.880, *r*_PC2_ = 0.832, *r*_PC3_ = 0.723). With one exception, the PCs show little variation in Europe and East Asia. That exception is PC3, which separates the Tundra Nentsi from the other East Asian populations and groups them with the Native American populations. PCs 4–6 account for an additional 1.6% of the variation. They capture substructure in the African populations, separating the San, Mbuti and Biaka from other African populations. The Native American populations are largely invariant for these three PCs. These results provide additional evidence of the pervasive influence of African and European admixture on patterns of Native American genetic diversity.

**Fig 6:**
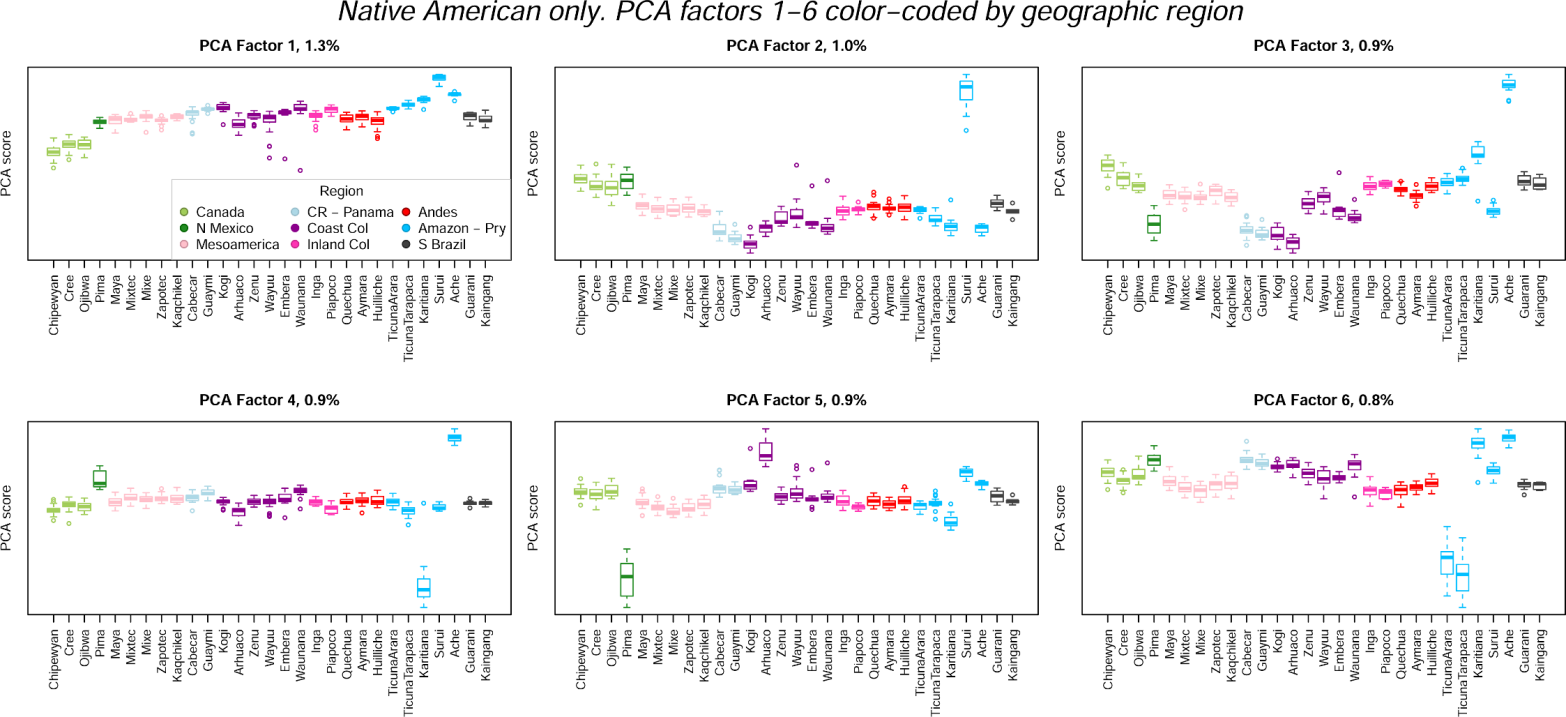
Boxplots of PCAs 1–6 for analyses of the Native American populations only, color-coded by geographic region.

We repeated the PCA after removing African, European and East Asian populations. Boxplots of the first six PCs from this analysis are shown in Fig 6. These PCs account for 5.7% of the variation. PC1 is strongly correlated with PC1 from the 63-population analysis (*r* = 0.92), and therefore with European ancestry. Subsequent PCs capture substructure within the Americas, associated mainly with the Amazonian and Paraguayan populations. Mesoamerican and Andean populations have similar scores on PCs 2–10, only separating from one another for PC 11 (not shown), and only then because the Mixe and Mixtec populations separate from the other Mesoamerican populations.

### Are patterns of population genetic and linguistic variation correlated?

In the 29 Native American populations, there are 16 well-attested language families, with an average of 1.8 languages per family. Only two of the families have more than two population representatives: Chibchan (n = 4), and Tupi-Guaraní (n = 4).

**Fig 7:**
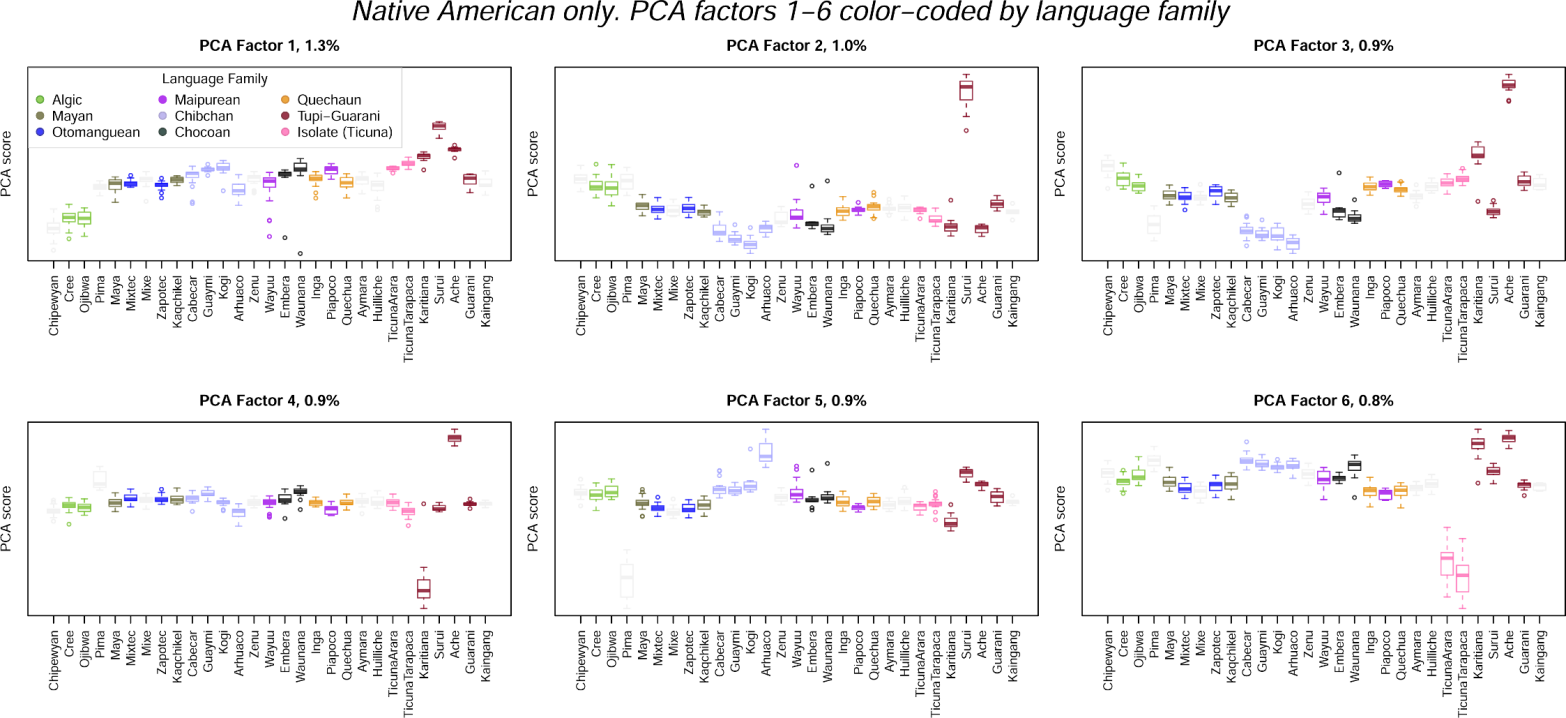
Boxplots of PCAs 1–6 for analyses of the Native American populations alone, color-coded by language family.

The PCs also provide information about the relationship between patterns of genetic and linguistic diversity in the Americas. Fig 7 shows PCs 1–6 from the Native-American-only analyses, this time color-coded by language family. The most notable linguistic structure is evident on PC 3, where the four Chibchan-speaking populations cluster together. Otherwise, none of the language families have distinctive scores for any of the PCs.

**Fig 8:**
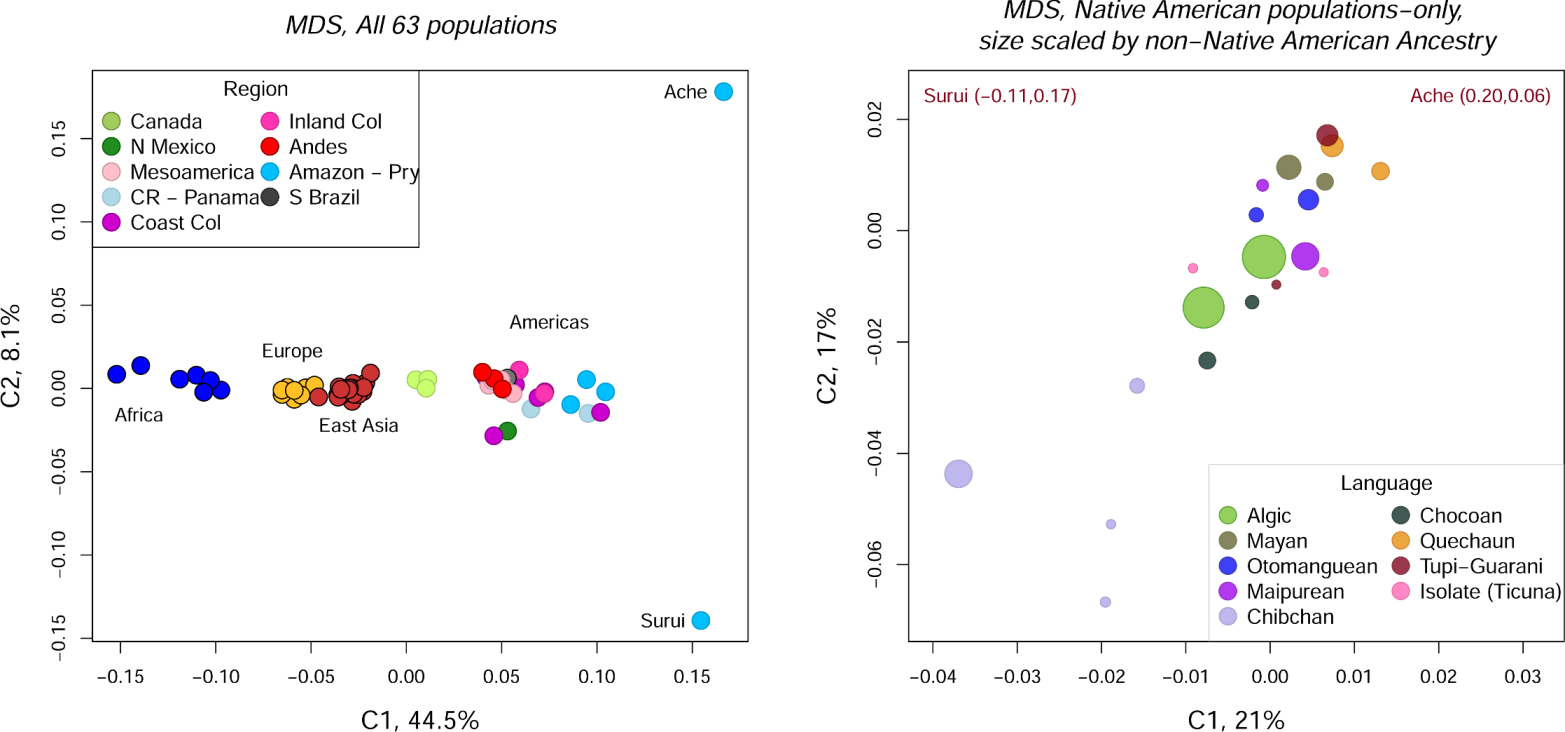
MDS plots of Nei’s minimum genetic distances. A. All 63 populations, color-coded by geographic region. B. 29 Native American populations only, color-coded by language family and sized by the level of non-Native American ancestry. The Tupi-Guaraní-speaking Ache and Suruí populations fall well outside of the plot margins. Their PCo scores are shown in the upper right and left portions of the plot.

We also constructed MDS plots from Nei’s minimum genetic distances. Fig 8A shows the MDS plot for the 63-population sample. PCo1 separates the major geographic regions. The Native American PCo1 scores are strongly correlated with European ancestry (*r* = -0.649). PCo2 distinguishes the Ache and Suruí from the other Native American populations. Consistent with the PCA analyses described above, the plot shows that Andean populations (in red) have relatively short genetic distances to Mesoamerican populations (in light pink).

Fig 8B is an MDS plot constructed from the Native American genetic distances only. The populations are color-coded by language family, and the point size is scaled by the proportion of non-Native American ancestry. The plot shows that, even though populations from the same language family tend to have low genetic distances, they are frequently even less distant to populations from different families. The Chibchan-speaking populations are the lone exception. However, even in this case, the Chibchan-speaking Cabécar in Costa Rica are genetically closer to the Chocoan-speaking Emberá population than they are to the other Chibchan-speaking populations. These results indicate that there is, at best, a weak association between genes and languages at the large geographic and linguistic scale covered by the populations in this analysis.

## Discussion

Our inferences about Native American prehistory were based in part on the choice of *K* in the *STRUCTURE* analyses. In many studies, the value of *K* is chosen based on the posterior probability of the data for a given *K*, P(*X*|*K*). In our analyses of the 63-population dataset, *K* = 9 had the highest posterior probability. Evanno et al. [30] demonstrated using simulations that 1) P(*X*|*K*) plateaued or continued to increase beyond the true value of *K*, and 2) the variance in this probability across runs (at a given value of *K)* increased beyond the true value. Based on these observations, the authors determined that a statistic related to the second order rate of change in the estimate of the posterior probability, termed Δ*K*, peaked at the true value of *K*. This approach performed well in simulations of several models of evolution, including a hierarchical island model consisting of sub-populations nested inside of larger demes, even when one deme was excluded from the analysis. This model is a reasonable representation of the structure of human diversity [27].

In our analyses of the 63-population dataset, ΔK also peaked at *K* = 9. At this and higher values of *K*, estimates of European and East Asian ancestry were much lower than at values of *K* from 5 to 8. Independent of P(X|*K*) and ΔK, sensible geographically-based genetic substructure continued to form at values of *K* well above 9. These results imply that some previous studies of admixture in Native Americans may have overestimated European and East Asian ancestry. In supervised runs of *STRUCTURE* at *K* = 5, for example, Wang et al. [1] identified a north-south gradient of Siberian (Yakut and Tundra Nentsi) ancestry in the Americas. In the current study, at *K* = 9 and higher, East Asian ancestry was low across the Americas, and it was uncorrelated with geographic distance from Beringia.

In a related vein, a recent cluster analysis of autosomal SNP diversity by Riech et al. [4] found that roughly 40% of the ancestry in the Na-Dene-speaking Chipewyan population was Siberian in origin. This finding might reflect the fact that the authors analyzed 17 Siberian populations, in contrast to the two Siberian populations analyzed in our study. However, they conducted their analysis at K = 4, so it is also possible that the Siberian component that they identified in the cluster analysis was really comprised of one or more Native American-specific clusters. This latter interpretation is consistent with the fact that, using more sophisticated statistical methods, Reich and colleagues also found that members of the Chipewyan population derived about 10% of their genomes from a second-wave of migration into the Americas. In our analysis, the signal of this second wave of migration could be contained within the Chipewyan-specific genetic cluster that we identified at higher values of K.

In unsupervised *STRUCTURE* runs of the same data, Hunley and Healy [12] identified a north-south gradient of European ancestry in the Americas. They reported especially high levels of European ancestry in the three Native Canadian populations and concluded that the decay in diversity from Beringia was in fact the product of geographically patterned admixture with Europeans. In the current study, we found that that European ancestry in the Canadian populations also decreased substantially as *K* increased (though it remained high in the Cree and Ojibwa, and, overall, was strongly correlated with distance from Beringia). Further, in our partial correlation analysis, the strong negative correlation between gene diversity and geographic distance from Beringia persisted when total non-Native American ancestry was controlled. These results demonstrate that studies of evolution in admixed populations are sensitive to the choice of the value of *K*.

### What routes did populations take during their initial dispersal across the Americas?

While is tempting to view the strong correlations between gene diversity and distance from Beringia as evidence for a north-south serial founder effect process, our partial correlation analyses revealed that 1) gene diversity is highest in the Mesoamerican and Andean populations, not the Canadian populations, and 2) the correlation between gene diversity and geographic distance from Beringia loses statistical significance when the Amazonian and Paraguayan populations are removed from the analysis (Table 1). We emphasize that these results are not driven by our choice of paths of movement; results were similar for all paths, including direct great circle distances from Beringia. These results are potentially inconsistent with a straightforward north-south serial founder effect process.

That said, it is important to note that we cannot reject the hypothesis that a north-south serial founder effect process ever occurred in the Americas. We have attempted to control for the effects of European contact on patterns of Native American diversity, but many other complex evolutionary processes occurred subsequent to initial peopling, including continued movements and interactions between peoples in the Americas and East Asia [4,42], possible independent waves of migration into Americas [43], and long-range populations movements within the Americas [44]. These processes may have obscured evidence for an earlier founder effect process. Additionally, even though many of the regional patterns that we identified have been replicated in other studies that used different data (many of which we have cited in this study), it is possible that other samples and data might reveal a different history.

In our analyses, we also found that patterns of diversity point to close affinity between Andean and Mesoamerican populations, consistent with findings of previous studies [1,8,9]. As Wang and colleagues [1] noted, this close affinity may be the product of high rates of gene flow between Mesoamerican and Andean populations or rapid long distance dispersal along the western coast of the Americas [4,45,46]. In either case, Colombian populations located between Mesoamerica and the Andes appear to have been unaffected, whether located near the coast (Waunana) or in the Andes (Inga). Importantly, on its own, the high level of diversity in Andean populations is uninformative about the peopling of Amazonia, where genetic drift has played a dominant role in shaping diversity [8].

### Genes and languages

It is difficult to assess the extent of gene-language congruence in the Americas prior to European contact because that contact resulted in the deaths of millions of people and the extinction of hundreds of languages [47,48]. Even now, the rate of language extinction is alarming. Based on data from Krauss [47] and Foster [49], for example, Campbell (1997) predicted that 80% of Native North American languages would be extinct within a generation. In South America, Ribero [50] found that only 14% of Native Brazilian populations were isolated from neo-Brazilian population centers in 1957, compared to 46% in 1900. Further, 33 of the 105 Native Brazilian groups that were isolated in 1900 were extinct by 1957. For these reasons, studies of the association between patterns of linguistic and genetic diversity in the Americas are unlikely to be informative in general about gene-language coevolution. Even setting aside the disruptive effects of European contact, gene-language congruence is unlikely to exist at the large geographic and linguistic scale analyzed here because 1) languages change quickly, erasing any phylogenetic signal that once existed, and 2) genes and languages move independently between populations.

To the extent that the current distribution of Native South American languages is in any way representative of the pre-contact period, the only language family that was genetically cohesive in the current study was Chibchan, which has a limited geographic distribution compared to other South American languages [25]. However, even here, the congruence was imperfect. Based on these results, we conclude that there is scant evidence for the co-evolution of genes and languages in this sample.
